# Basic Musculoskeletal Imaging

**Published:** 2017-03

**Affiliations:** Department of Orthopaedics, Universiti Sains Malaysia, Kubang Kerian, Malaysia

Radiological imaging has become a routine assessment for patients with orthopaedic and traumatic disorders. Diagnosis and treatments of orthopaedic conditions would not be commenced safely prior to appropriate radiological assessment. At times, orthopaedic surgeons need to be independent and not relying too much on their radiological colleagues, as many conditions can be easily interpreted. It is not practical to consult a radiologist for all cases. In the era of disproportionately high medico legal incidence, we cannot afford to miss a diagnosis. Orthopaedic surgeons must have the basic understanding of radiological imaging and must at certain degree, be able to interpret them. However, complex orthopaedic disorders including musculoskeletal malignancy, spinal and paediatric conditions still require assistance from a trained radiologist.

*Basic Musculoskeletal Imaging* by Jamshid Tehranzadeh is a good book to start with for those who want to understand and interpret radiological images. It is simple, concise and covers most of the important topics in orthopedics. The topics include skeletal trauma of upper and lower limbs, pediatric skeletal trauma, arthritis, infections, tumour, metabolic bone diseases, bone infarct, osteochondrosis, orthopaedic hardware and special coverage on MRI of the joints. There is a special topic on signs in musculoskeletal radiology (Chapter 11). There are 20 chapters in this book and written by experts in the fields.

The first chapter covers imaging modalities used in musculoskeletal radiology. The author discusses the principles of radiographs, computed tomography, ultrasound, MRI, nuclear medicine, bone scan and PET scan. It gives a clear understanding of all the imaging techniques in simple explanation. It is not too long that might bore the nonradiologist trained in the background (That means us)!). This chapter forms the basis of understanding for the subsequent chapters of the book.

As the title implies, this book gives the basic introduction and understanding of musculoskeletal imaging. But don’t be surprised that it doesn’t only cover basic radiographs, it discusses other *non-basic* radiological imaging including musculoskeletal ultrasound and scintigraphy as well. At the end of every chapter, the summary of important facts are given in “Pearls”. However, don’t expect too many details and complicated physics or mathematical formulae from it. Nowadays, complex radiological investigations are performed on patients. MRI for instance is so routinely done on patients for its sensitivity and specificity. Special topics on MRI of the joints (7 different chapters covering all the major joints in the body) is accommodating because it is most relevant and tricky for most of us, especially the surgeons in training.

The authors had attempted to address common musculoskeletal disorders seen in daily practice and providing the relevant imaging and explanation, in the simplest possible way. Important radiological signs were highlighted for the readers to recognize and understand them. However the readers should be aware that, the imaging are probably the typical ones and not seen in all cases. They should be able to recognize and look hard for them in the images of their patients.

I found this book interesting and helpful because it covers most of the common issues in orthopedics imaging. This is a collection of most common orthopaedic imaging seen in practice. The images are excellent and most of the relevant information needed is available in the book. The book is quite handy for quick reference.

This book is recommended for those who are committed to improve their radiological diagnostic abilities and standard of patients’ care. It may provide assistance to those who want to understand the basic principles of radiological imaging of musculoskeletal system. This book is certainly useful for orthopaedic and radiologists in training. It is also useful for senior consultants who want to teach the inexperienced orthopaedic fellows in training. But don’t ever think this book will replace the need to consult a trained radiologist when it comes to complicated radiological images.

